# Unraveling the gut‐brain connection: The association of microbiota‐linked structural brain biomarkers with behavior and mental health

**DOI:** 10.1111/pcn.13655

**Published:** 2024-02-29

**Authors:** Oren Contreras‐Rodriguez, Gerard Blasco, Carles Biarnés, Josep Puig, Maria Arnoriaga‐Rodríguez, Clàudia Coll‐Martinez, Jordi Gich, Lluís Ramió‐Torrentà, Anna Motger‐Albertí, Vicente Pérez‐Brocal, Andrés Moya, Joaquim Radua, José Manuel Fernández‐Real

**Affiliations:** ^1^ Department of Radiology‐Medical Imaging (IDI), Girona Biomedical Research Institute (IdIBGi) Dr. Josep Trueta University Hospital Girona Spain; ^2^ Department of Psychiatry and Legal Medicine, Faculty of Medicine Universitat Autònoma de Barcelona Bellaterra Spain; ^3^ Health Institute Carlos III (ISCIII) Madrid Spain; ^4^ CIBERSAM Madrid Spain; ^5^ Radiology Department CDI Hospital Clinic of Barcelona Barcelona Spain; ^6^ Department of Diabetes, Endocrinology, and Nutrition (UDEN), Girona Biomedical Research Institute (IdIBGi) Dr. Josep Trueta University Hospital Girona Spain; ^7^ CIBER Fisiopatología de la Obesidad y Nutrición (CB06/03/0010) Girona Spain; ^8^ Neuroimmunology and Multiple Sclerosis Unit, Department of Neurology Dr. Josep Trueta University Hospital Girona Spain; ^9^ Department of Medical Sciences, School of Medicine University of Girona Girona Spain; ^10^ Department of Genomics and Health Foundation for the Promotion of Health and Biomedical Research of Valencia Region (FISABIO‐Public Health) València Spain; ^11^ CIBEResp Madrid Spain; ^12^ Institute for Integrative Systems Biology (I2SysBio) The Spanish National Research Council (CSIC‐UVEG), The University of Valencia València Spain; ^13^ Imaging of Mood‐ and Anxiety‐Related Disorders (IMARD) Group Institut d'Investigacions Biomèdiques August Pi i Sunyer (IDIBAPS) Barcelona Spain; ^14^ Department of Medicine, Faculty of Medicine and Health Sciences University of Barcelona Barcelona Spain

**Keywords:** brain structure, cognition, depression, gut microbiota, machine‐learning

## Abstract

**Aim:**

The gut microbiota can influence human behavior. However, due to the massive multiple‐testing problem, research into the relationship between microbiome ecosystems and the human brain faces drawbacks. This problem arises when attempting to correlate thousands of gut bacteria with thousands of brain voxels.

**Methods:**

We performed brain magnetic resonance imaging (MRI) scans on 133 participants and applied machine‐learning algorithms (Ridge regressions) combined with permutation tests. Using this approach, we were able to correlate specific gut bacterial families with brain MRI signals, circumventing the difficulties of massive multiple testing while considering sex, age, and body mass index as confounding factors.

**Results:**

The relative abundance (RA) of the *Selenomonadaceae*, *Clostridiaceae*, and *Veillonellaceae* families in the gut was associated with altered cerebellar, visual, and frontal T2‐mapping and diffusion tensor imaging measures. Conversely, decreased relative abundance of the *Eubacteriaceae* family was also linked to T2‐mapping values in the cerebellum. Significantly, the brain regions associated with the gut microbiome were also correlated with depressive symptoms and attentional deficits.

**Conclusions:**

Our analytical strategy offers a promising approach for identifying potential brain biomarkers influenced by gut microbiota. By gathering a deeper understanding of the microbiota‐brain connection, we can gain insights into the underlying mechanisms and potentially develop targeted interventions to mitigate the detrimental effects of dysbiosis on brain function and mental health.

Preclinical studies have accumulated evidence that manipulations of the gut microbiota can modulate emotion, cognition, and associated behaviors.^1^, ^2^, ^3^ The administration of probiotics as a potential treatment for neurological and psychiatric disorders in humans has been investigated in both experimental and clinical studies.^4^ However, there is a paucity of data on the relationship between human gut microbiome ecosystems and the brain,^5^ with few neuroimaging studies demonstrating an association between gut microbiome and magnetic resonance imaging (MRI) signals in healthy and pathological conditions.^2^, ^3^, ^6^, ^7^, ^8^, ^9^, ^10^, ^11^, ^12^ Suitable analysis algorithms are required because searching for potential connections between a determined microbiome ecosystem and the thousands of brain voxels entails a huge multiple‐testing problem. For instance, we would need to conduct nearly half a billion tests to test the relationship between each ~400 microbiota families and each ~900,000 voxels of a standard MRI image. Neuroimaging studies commonly address this problem by correcting for multiple comparisons across voxels. Still, with this approach, we should conduct hundreds of tests, as we would need a separate test for each family, which entails multiple testing issues.^13^ Therefore, a method capable of testing the relationship between all bacterial ecosystems in all voxels simultaneously is required. However, such a method has yet to be developed.

In addition, integrating MRI modalities with bacterial microbiome ecosystems may improve our ability to identify biologically significant brain biomarkers.^14^ Identification of brain biomarkers for Alzheimer disease,^15^ mild cognitive impairment,^16^ Parkinson disease,^17^ and subtypes of progressive aphasia^18^ have already demonstrated the potential of multimodal MRI. From a structural point of view, employing diverse brain imaging modalities enhances the possibility of revealing the structural brain features most associated with the gut microbiota. For example, T1‐weighted anatomical sequences are routinely used as a noninvasive tool to derive measurements of gray matter (GM) loss (lower values) or gain (higher values).^19^ In turn, diffusion tensor imaging (DTI) is well suited for visualizing the microstructural details of white matter in vivo using several metrics.^20^ DTI‐related metrics include fractional anisotropy (i.e. a summary measure of microstructural integrity or other phenomena), mean diffusivity (MD; i.e. the overall magnitude of water diffusion), and axial diffusivity (AD; i.e. a measure of water diffusion along the principal axis of diffusion) and radial diffusivity (RD; i.e. a measure of water diffusion along the perpendicular axis), considered as potential in vivo surrogate markers of axonal and myelin damage.^21^, ^22^ Higher values in T2‐mapping sequences are associated with histologic brain features such as gliosis, neuronal loss, or extracellular space expansion.^20^, ^23^

The current study aims to explore potential associations between multiple structural MRI modalities (T1, DTI, T2 mapping) and the gut microbiota at the level of bacterial families in 133 adults while controlling for sex, age, and body mass index (BMI). Using a multivariate approach, we developed a new strategy to overcome the multiple tests derived from the massive number of brain voxels and bacteria. This entails predicting the value of a given voxel on the relative abundances (RAs) of all families of bacteria at once by fitting machine‐learning algorithms (Ridge regressions) to conduct a single analysis per voxel and, afterward, correcting for multiple testing across voxels. After identifying the voxels in which MRI measurements showed an overall relationship with the microbiota RA, we investigated the contribution of the specific bacteria and their correlation with cognitive performance and depressive symptoms.

## Methods

### Participants

From January 2016 to October 2017, 133 individuals participated in a study conducted by the Endocrinology Department of Dr. Josep Trueta University Hospital. Participants of both sexes older than 18 years were eligible. Exclusion criteria were as follows: (i) presence of current or past medical illness (e.g. metabolic disease, cancer, inflammatory‐related illnesses) or incapacitating psychiatric disorders (e.g. severe eating disorder or major psychiatric antecedents including major depression), as evidenced by semistructured interviews, except for obesity (BMI = weight/(height)^2^); (ii) MRI contraindications (e.g. claustrophobia, ferromagnetic implants); (iii) excessive acute or chronic alcohol intake (i.e. ≥ 40 g alcohol (OH)/day in women or ≥ 80 g OH/day in men); (iv) clinical symptoms and signs of infection in the previous month or antibiotic, antifungal, or antiviral treatment in the previous 3 months; and (v) pregnancy and lactation. The study protocol was approved by the institutional review board ethics committee and the Committee for Clinical Research at the University Hospital of Girona Dr. Josep Trueta in Girona, Spain. All procedures complied with the institutional and national committees’ (responsible for human experimentation) ethical standards and the 1975 and 2008 versions of the Declaration of Helsinki. All participants provided informed written consent before the start of the study.

### Acquisition and preprocessing of MRI data

All participants underwent an MRI scanning session in a 1.5‐T Ingenia system (Philips Healthcare) with 15‐channel head coils. The session included a T1 anatomical sequence (repetition time [TR] = 8.3 ms, echo time [TE] = 4.1 ms, flip angle = 8°, field of view [FOV] = 230 × 190 mm, 232 × 229 pixel matrix; slice thickness = 1 mm); a DTI sequence (single‐shot echo planar imaging sequences with the sensitivity encoding parallel imaging scheme‐acceleration factor 2‐, 15 noncollinear directions with a b value of 1000 s/mm^2^, TR = 6795 ms, TE = 72 ms, FOV = 230 × 230 pixel matrix, voxel size 2 × 2 × 3 mm^3^, 32 sections, 3 min 10 s acquisition time); and a T2 mapping (TR = 2000 ms, TE = 15 + n × 20 ms [five echoes], FOV = 230 × 230 mm, 144 × 138 pixel matrix; slice thickness = 5 mm).

We processed and analyzed the MRI data with MATLAB version R2017a (The MathWorks Inc.) and Statistical Parametric software (SPM12; The Welcome Department of Imaging Neuroscience) unless otherwise specified. T1 images were segmented using the “new segment” algorithm. The rigidly transformed versions of GM images derived from this algorithm were normalized using a Diffeomorphic Anatomical Registration Through Exponentiated Lie Algebra (DARTEL) algorithm.^24^ Participants’ native space GM images were registered to the highest resolution GM template within a high‐dimensional diffeomorphic framework, modulated by the Jacobian determinants of the corresponding flow fields, registered to the standard SPM template, and resliced to 2‐mm resolution. We used Olea Sphere 3.0 (Olea Medical) to check the image quality, correct signal, and motion distortions. After this inspection, we discarded six T1 sequences, eight DTI sequences, and seven T2‐mapping sequences due to acquisition or movement artifacts. We subsequently computed the common scalar maps from DTI (i.e. fractional anisotropy, MD, AD, RD), and T2 mapping, and applied the skull stripping method with the brain extraction tool (BET). Then, we used in‐house templates of each parametric map made from a representative healthy cohort and registered to the standard SPM template at a 2‐mm resolution to conduct the spatial normalization. We smoothed all T1, T2 mapping, and DTI maps using an isotropic Gaussian kernel with a full width at half‐maximum of 8 mm.

### Extraction of fecal genomic DNA and genome sequencing

Stool samples from the study participants were obtained and preserved at −80°C. Total DNA was extracted from frozen human stool using the QIAamp DNA Stool Mini Kit (QIAGEN) following the manufacturer's instructions. Quantification of DNA was performed with a Qubit 3.0 fluorometer (Thermo Fisher Scientific), and 1 ng of each sample (0.2 ng/μL) was used for shotgun library preparation for high‐throughput sequencing, using the Nextera DNA Flex Library Prep kit (Illumina, Inc.) according to the manufacturer's protocol. Sequencing was performed on a NextSeq 500 sequencing system (Illumina) with 2 × 150‐bp paired‐end chemistry, at the facilities of the Sequencing and Bioinformatic Service of the FISABIO. The obtained input FASTq files were decompressed, filtered, and 3′ ends‐trimmed by quality, using the prinseq‐lite‐0.20.4 program and overlapping pairs were joined using FLASH‐1.2.11. Fastq files, converted into fasta files, were mapped against the reference human genomes (GRCh38.p11, December 2013 and GRCm38.p6, September 2017), respectively, to remove reads from host origin, by using bowtie2‐2.3.4.3 with end‐to‐end and very sensitive options.

### Depressive symptoms and cognitive assessments

We assessed depressive symptoms using the Patient Health Questionnaire 9 (PHQ‐9).^25^ This questionnaire has nine items that range from 0 to 27. Scores of 5, 10, 15, and 20 represent cut points for mild, moderate, moderately severe, and severe depressive symptoms, respectively. We used a comprehensive battery of neuropsychological tests, including the Stroop Color and Word interference test,^26^ the Trail Making Test (TMT),^27^ and the Total Digit Span Task (DST) to assess cognition. The Stroop test assesses inhibitory control as a measure of the identification of the color ink of incongruent color‐word stimulus (STROOP‐CW). The TMT measures attention, processing speed, and mental flexibility. It requires the participant to connect in proper order 25 numbers randomly disposed on the paper (TMT‐A) or 25 numbers and letters in alternating order (TMT‐B) also randomly arranged on the paper as quickly as possible. In this case, more score (seconds), less performance. The DST is a subtest of the Weschler Adult Intelligence Scale III (WAIS‐III),^28^ a measure of working memory function, which also includes the Forward (i.e. working memory and attention) and Backward (i.e. working memory) Digit Span. The total DST represents the total score of previous tests.

### Statistical analysis

Given the vast number of brain voxels and bacteria families, the number of correlation tests between each voxel and the RA of each bacteria family would be massive, preventing any sensible control of multiple testing. To overcome this limitation, we used a multivariate approach that reduced the number of tests. Specifically, we fitted machine‐learning algorithms to predict the value of a given voxel from the RA of all families of bacteria at once, thus conducting one single analysis per voxel. Afterward, we corrected for multiple testing across the voxels using standard neuroimaging permutation tests. We detail the procedures in the following.

First and separately for each voxel, we conducted a Ridge regression of the voxel value as a function of the (logarithm‐transformed, see next) RA of all microbiota families, plus the covariates (sex, age, and BMI). We previously replaced any RAs <10^−3^ with 10^−3^ to avoid outlying effects of tiny RAs (with their logarithm‐transforms approaching minus infinity) and limited the analysis to those families in which at least 5% of individuals had RAs >1% and those voxels in which the amount of tissue or signal was moderately large to avoid findings in implausible locations (e.g. outside the brain). Ridge regression is a multiple regression method that accepts multicollinear regressors, as could be the case of microbiome families. Specifically, the method improves efficiency in estimating the coefficients in exchange for tolerably biasing them downward.^29^ Notably, we were interested in detecting patterns of RA among sets of families (e.g. whether MRI signals were associated with “higher RA of family A than of family B” rather than “high RA of family A and/or low RA of family B”) and potential nonlinear relationships in the patterns (e.g. quadratic curves). Given that the natural logarithm can easily accommodate these patterns, these aims led us to apply it to the RA. For instance, if the regression estimates β_A_ to be ≈1 and β_B_ to be ≈−1, then exp(y) = RA_A_/RA_B_. Also, if it estimates β_A_ to be ≈2, then exp(y) = RA_A_^2^. Similarly, if it estimates β_A_ to be ≈0.5 and β_B_ to be ≈−0.5, then exp(y) = sqrt(RA_A_/RA_B_).

To measure the association between the microbiota and the voxel, we used the RSS Ratio, which indicates the improvement in the residual sum of squares (RSS) when conducting the Ridge regression with the microbiota and the covariates, as compared with a Ridge regression with only the covariates:

$$\mathrm{RSS}\;\text{Ratio}=\frac{\mathrm{RS}S_{\text{covariates}}}{\mathrm{RS}S_{\text{microbiota and covariates}}}$$

Importantly, to avoid overfitting, we calculated the RSS using a 10‐fold cross‐validation approach. Specifically, we used nine tenth parts of the participants to train the Ridge regressions. Then, we calculated the RSS in the tenth part of the participants we had not used to train the regression. We then repeated the process with another tenth part of the participants, and so on, until we had calculated the RSS of all participants.

Second, we conducted a Freedman‐Lane permutation test^30^ to derive the *P* values of the RSS ratios, both uncorrected (for exploratory analysis) and family‐wise error (I) corrected with threshold‐free cluster enhancement^31^ for multiple testing across voxels. The permutation test was extremely computer‐demanding, for which we first only conducted 50 permutations and, afterward, 200 extra permutations for those modalities with potential associations. We primarily report findings with FWE <0.05 (i.e. corrected for multiple testing). However, for the sake of exhaustivity, we also report findings with uncorrected *P* < 0.004 (i.e. the minimum *P* value with 250 randomizations) as long as the RSS ratio was >1.33 (i.e. a > 25% reduction of the RSS when including the microbiota in the Ridge regression).

We recalculated the RSS ratios for the voxels significantly associated with the RA of families of bacteria in the following subgroup analyses: only individuals with overweight/obesity (BMI > 25), only participants who had at least one yogurt per week as evaluated through a validated food frequency questionnaire,^32^ only nonsmokers, only individuals with low alcohol intake (<10 g per day), only participants not taking antidepressants, and only individuals not using anxiolytics. We could not conduct these analyses with normal‐weighted individuals or participants having less than a yogurt a week because there were too few (*n* = 27 and *n* = 26, respectively).

Finally, we further investigated those voxels in which MRI measures showed an overall relationship with the microbiota RA. On the one hand, we extracted the Ridge regression coefficients to evaluate each family's contribution. On the other hand, we correlated the clinical variables (depressive symptoms and cognitive measurements) with the MRI signal, using the false discovery rate method to correct for multiple testing.

We conducted all calculations in R (R Foundation) with the packages “oro.nifti,”^33^ “doParallel,”^34^ and “ridge.”^35^

## Results

The characteristics of the study participants, including main demographics, health information, and cognitive performance, are listed in Table 1.

**Table 1:** Demographic, health, and cognitive information of the 133 study participants

	Statistic	Range (minimum–maximum)
Age (years)	46.68 (10.65)	22–66
Sex (female), n (%)	92 (69.2)	‐
Education (years)^†^	13.60 (3.58)	1–20
BMI (kg/m^2^)	35.31 (10.88)	19.2–63.4
Smoking (yes), n (%)	63 (47.3)	‐
Alcohol intake (g/day)^‡^	3.72 (6.78)	0–37.2
Antidepressant (yes), n (%)	33 (24.8)	‐
Anxiolytics (yes), n (%)	20 (16.3)	‐
PHQ‐9^§^	6.31 (4.39)	0–22
Total Digit Span^¶^	14.69	7–29
Digits Forward^¶^	8.29	4–13
Digits Backward^¶^	6.32	2–14
TMT‐A^¶^	32.44	13–73
TMT‐B^¶^	72.78	0–236
STROOP‐CW^††^	43.23	17–70

Mean and standard deviations (SDs) are provided, except where otherwise indicated.

BMI, body mass index; PHQ‐9, Patient Health Questionnaire 9, used to assess depressive symptoms; STROOP‐CW, Stroop Color and Word Test, used to assess inhibitory control; TMT, Trail Making Test, used to assess attention, processing speed, and mental flexibility.

^†^ *N* = 126 participants.

^‡^ *N* = 129 participants.

^§^ *N* = 124 participants.

^¶^ *N* = 124 participants.

^††^ *N* = 123 participants.

### Relationship between brain and bacterial gut microbiota

#### T2 mapping

**Fig. 1:**
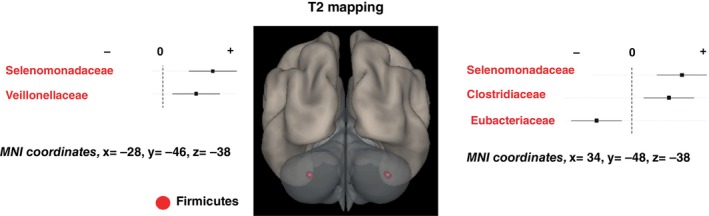
Graphic display of the bilateral cerebellum in which the T2‐mapping signal showed an association with the bacterial families. Bacterial families driving the relationships at a *P* < 0.01 are indicated in the corresponding plots and colored according to phylum. The right hemisphere corresponds to the right side of the coronal view. MNI, Montreal Neurological Institute.

The only finding that survived FWE <0.05 in the Ridge regression analysis was a relationship between the RA of some bacterial families and the T2 values in the right cerebellum, but we also found subthreshold associations bilaterally with two other regions of the cerebellum/fusiform gyrus, as well as the right medial gyrus rectus and the left posterior occipital cortices (Table 2). Specifically, increased RAs of bacterial families of the phyla Firmicutes (i.e. *Selenomonadaceae*, *Veillonellaceae*, *Clostridiaceae*), Bacteroidota (i.e. *Odoribacteraceae*), and Bacillota (i.e. *Oscillospiraceae*), and decreased RAs of the bacterial family *Eubacteriaceae* were associated with higher T2 values (Table 2). Figure 1 shows the prominent association between the RA of the bacterial families and the most significant T2 values coordinates in the right and left cerebellum. Figure S1 provides a complete map of associations between the RA of the bacterial families and the T2 values.

**Table 2:** Relationships between the relative abundance of microbiome families and structural T2 mapping and DTI brain metrics

					Main specific families related	
	x, y, z	RSS ratio	*P* value	CS	Positive	Negative
T2‐mapping signal						
Cerebellum (Cr I‐II)	34, −48, −38	1.611	FWE <0.05	23	*Selenomonadaceae* **	*Eubacteriaceae* *
					*Clostridiaceae* *	
	−28, −46, −38	1.460	0.004	21	*Selenomonadaceae* **	
					*Veillonellaceae* *	
Inferior occipital	−32, −82, −12	1.427	0.004	6	*Odoribacteraceae* **	
					*Oscillospiraceae* *	
Fusiform gyrus	36, −34, −30	1.368	0.004	2	*Veillonellaceae* *	
Medial gyrus rectus	0, 44, −24	1.349	0.004	1	*Selenomonadaceae* ***	
					*Veillonellaceae* *	
Cerebellum (Cr I‐II)	−46, −56, −36	1.342	0.004	1	*Streptococcaceae* ***	
					*Veillonellaceae* **	
Axial diffusivity						
Middle frontal gyrus	56, 22, 40	1.393	0.004	2	*Selenomonadaceae* *	*Sutterellaceae* **
					*Veillonellaceae* *	*Bifidobacteriaceae* *
Dorsomedial frontal cortex	−4, 26, 50	1.336	0.004	1	*Selenomonadaceae* **	*Sutterellaceae* *
					*Veillonellaceae* *	
Inferior frontal gyrus/insula	−48, 14, 6	1.369	0.004	2	*Selenomonadaceae* ***	
					*Streptococcaceae* ***	
Mean diffusivity						
Middle frontal gyrus	56, 22, 40	1.358	0.004	2	*Selenomonadaceae* **	*Sutterellaceae* **
Dorsomedial frontal cortex	−4, 28, 52	1.364	0.004	2	*Selenomonadaceae* **	*Sutterellaceae* *
					*Veillonellaceae* *	
					*Bacteroidaceae* *	
Radial diffusivity						
Middle frontal gyrus	56, 22, 40	1.342	0.004	2	*Selenomonadaceae* **	*Sutterellaceae* **
Dorsomedial frontal cortex	−4, 28, 50	1.366	0.004	3	*Selenomonadaceae* **	*Sutterellaceae* *
					*Bacteroidaceae* *	

Coordinates (x, y, z) are in the Montreal Neurological Institute (MNI) Atlas space. Significance of the associations with specific families.

CS, cluster size; DTI, diffusion tensor imaging; FWE, familywise error rate; RSS ratio, improvement in the residual sum of squares (RSS) when conducting the Ridge regression with the microbiota and the covariates compared with a Ridge regression with only the covariates.

* *P* < 0.01.

** *P* < 0.001.

*** *P* < 10^−6^.

#### Diffusion Tensor Imaging

**Fig. 2:**
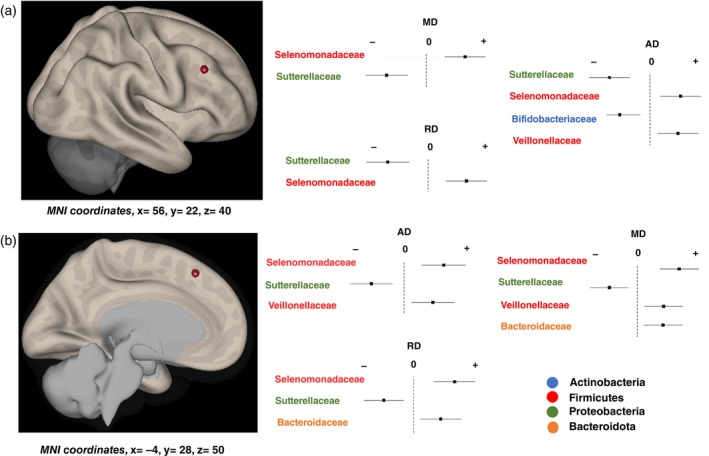
Graphic display of the middle frontal gyrus (a, right sagittal view) and the dorsomedial prefrontal cortex (b, left sagittal view) in which the axial diffusivity (AD), mean diffusivity (MD), and radial diffusivity (RD) diffusion tensor imaging (DTI) signals showed an association with the bacterial families. Bacterial families driving the relationships at a *P* < 0.01 are indicated in the respective plots and colored according to phylum. MNI, Montreal Neurological Institute.

The Ridge regression analyses revealed subthreshold associations between the RA of some bacterial families and the DTI‐related values (AD, MD, RD) in the right middle frontal gyrus and the left dorsomedial frontal cortex (Table 2, Fig. 2), while we observed a specific association for the AD signal in a region encompassing the left inferior frontal gyrus and the insula (Table 2). We found no significant associations with fractional anisotropy values. As for the T2 mapping, increased RA of bacterial families of the phyla Firmicutes (i.e. *Selenomonadaceae* and *Veillonellaceae*) were associated with higher DTI‐related values. Additional associations between the RA of the bacterial families and DTI‐related values included a positive association with the phyla Bacteroidota (i.e. *Bacteroidaceae*) and Bacillota (i.e. *Streptococcaceae*), as well as negative associations with the phyla Actinobacteria (i.e. *Bifidobacteriaceae*) and Proteobacteria (i.e. *Sutterellaceae*). Notably, the RA of *Sutterellaceae* showed an overall negative association with all DTI‐related values in the right middle frontal gyrus and the left dorsomedial prefrontal cortex (Table 2, Fig. 2). Figure S2 provides a complete map of associations between the RA of the bacterial families and the DTI‐related values.

These results were similar when only including participants with overweight/obesity (RRS ratios only slightly lower, −0.078 on average), when only including participants having at least one yogurt per week (RRS ratios only slightly lower, −0.108 on average), when only including nonsmokers (RSS ratios only marginally lower, −0.061 on average), or when only including participants with no/low alcohol intake (RSS ratios only marginally lower, −0.075 on average). The RRS ratios were smaller when only including individuals not taking antidepressants or when only including participants not using anxiolytics (RSS ratios −0.20 and −0.17 smaller on average).

### Associations between bacterial‐related brain values and clinical assessments

Table 3 displays the clinical variables related to the MRI measures of the voxels identified in the Ridge regression analyses (note that for DTI, we only included AD because MD and RD findings were nearly identical to AD findings). Primarily, the presence of potential attentional deficits (evaluated with TMT‐A) showed a positive relationship with AD values in the right middle frontal gyrus (*r* = 0.33, false discovery rate <0.05). We also observed trends (*P* < 0.05) of positive relationships between depressive symptoms (evaluated with PHQ‐9) and T2 values in the bilateral cerebellum and AD values in the right middle frontal gyrus, and negative correlations between these regions and STROOP‐CW scores.

**Table 3:** Relationship between the gut microbiota‐related brain peaks, cognition, and depressive symptoms

	x, y, z	Positive (correlation)	Negative (correlation)
T2‐mapping signal			
Cerebellum (Cr I‐II)	34, −48, −38	PHQ‐9 (*r* = 0.23)	
	−28, −46, −38	PHQ‐9 (*r* = 0.26)	STROOP‐CW (*r* = −0.23)
Cerebellum (Cr I‐II)	−46, −56, −36		
Axial diffusivity			
Middle frontal gyrus	56, 22, 40	TMT‐A (*r* = 0.33)*	STROOP‐CW (*r* = −0.24)
		PHQ‐9 (*r* = 0.23)	

Coordinates (x, y, z) are in the Montreal Neurological Institute (MNI) Atlas space.

PHQ‐9, Patient Health Questionnaire 9, used to evaluate depressive symptoms; STROOP‐CW, Stroop Color and Word Test, used to evaluate inhibitory control; TMT, Trail Making Test, used to evaluate attention, processing speed, and mental flexibility.

* False discovery rate <0.05; clinical variables without * survive *P* < 0.05.

## Discussion

We investigated potential associations between multiple brain structural MRI modalities (i.e. T2 mapping, DTI, T1) and the gut microbiota bacterial families in a sample of 133 individuals through a newly developed strategy based on Ridge regression analyses to overcome the multiple tests derived from the massive number of brain voxels and microbiota data. These analyses demonstrated that the RA of bacterial families was primarily associated with T2 and DTI values in the cerebellum, visual cortices (mainly for T2), and the frontal cortex. We found that the bacterial families from the phylum Firmicutes were positively associated with T2‐ and DTI‐related values in the cerebellum and medial and lateral prefrontal cortex areas (i.e. mainly *Selenomonadaceae* and *Veillonellaceae*). In addition, the *Sutterellaceae* family from the Proteobacteria phylum showed a recurrent negative association with DTI‐related values. Correlation analyses found positive associations between bacteria‐associated brain signals and depressive symptoms or attentional deficits. It is reasonable to wonder what these relationships could mean at this stage.

The significant relationships between the RA of some bacteria and the prefrontal cortex across MRI modalities are congruent with prior research.^2^, ^3^, ^36^, ^37^, ^38^, ^39^ Notably, our results agree with previously identified MRI changes in the medial and orbital sections of the prefrontal cortex in these studies. To our knowledge, no study has previously linked the composition of gut microbiota with the structural properties of the cerebellum. The association between the bacterial families and T2‐ and DTI‐related values is consistent with the evidence that the gut microbiota plays a role in specific changes in white matter architecture,^38^, ^40^ influencing the transcriptional activity of genes involved in neuronal myelination,^36^, ^37^ thereby contributing to chronic neuroinflammation and neurodegenerative pathology.^41^

This study designated the anaerobic phylum Firmicutes (*Selenomonadaceae* and *Veillonellaceae* families) as the bacteria most strongly associated with brain voxels. The characteristics of the study participants (e.g. presence of obesity) and the covariates considered may help to explain the differences between the studies. In the current study, the *Selenomonadaceae* and *Veillonellaceae* bacterial families showed the most prevalent positive association with T2‐ and DTI‐related values in the identified brain regions, including the prefrontal cortex and the cerebellum. Increases in T2 values have been associated with gliosis, neuronal loss, and expansion of the extracellular space. In contrast, increased AD, MD, and RD signals may indicate incipient structural loss within white matter tracts. Interestingly, members of the bacterial families *Veillonellaceae*, *Clostridiaceae*, *Eubacteriaceae*, *Bacteroidaceae*, and *Bifidobacteriaceae*, which we found to be associated with T2‐ and DTI‐related values, have been linked to cresol generation,^42^ and increased cresol levels in mice and exposure of oligodendrocytes to this metabolite prevented myelin gene expression and differentiation.^38^ We found only one study reporting a positive association between *Selenomonadaceae* and working memory.^3^ Additional research is required to comprehend the clinical and physiological significance of these bacterial families.

Other bacteria belonging to the phyla Proteobacteria (e.g. *Sutterellaceae*) were also associated with DTI‐related values. The phyla Proteobacteria harbors many pathogens,^43^ and it has been negatively associated with regional brain volumes^44^ and memory scores.^3^ These results may agree with the negative association found between this bacterial family and low diffusivity signals (i.e. AD, MD, and RD) in the dorsomedial prefrontal cortex and the middle frontal gyrus, which suggests the incipient alterations in the microarchitecture of the white matter tracts.

The significant links between the bacteria‐associated brain signals and depressive symptoms and cognitive processes can help to understand the potential impact on behavior. Depressive symptoms correlated positively with the T2 value of the cerebellum and the RD of the middle frontal gyrus. Certain bacteria associated with the brain signals in these regions herein (i.e. *Veillonellaceae*) are reduced in depression.^45^ The MD, AD, and RD in the middle frontal gyrus were positively associated with processing attentional speed. Notably, bacterial families of the phylum Actinobacteria (i.e. *Bifidobacterium*), uniquely associated with the AD signal of this region, showed a negative association with speed attention as assessed by the same TMT test in a previous study.^8^

It is important to acknowledge the limitations of this study. We devoted several months to the extremely computationally intensive calculations. As a result, we focused on identifying the fastest machine‐learning algorithm for the data rather than determining the most powerful one, and the statistical tests had fewer permutations than usual. Consequently, we cannot exclude the possibility that using different algorithms may have yielded findings with a higher statistical significance. Nonetheless, due to the exploratory nature of this study, we adopted a relatively lenient thresholding approach, which we believe enabled us to identify the most important relationships between gut microbiota and the brain. However, it is crucial to recognize that some of the identified associations may be spurious, and we caution readers to interpret our findings as preliminary until further studies confirm or refute them. A second limitation is that we used a DTI sequence with a limited number of directions. Future studies could use a higher number of directions in the DTI and smaller slice thickness in the T2‐mapping sequences to increase the accuracy of the obtained data. Also, we prioritized obtaining samples within a 4‐h window of fecal emission to ensure optimal preservation under favorable conditions. Still, it would have been desirable to focus on collecting microbiota samples at consistent times of the day due to the suggested bidirectional communication between the host's circadian rhythms and the gut microbiota.^46^ On the other hand, we did not investigate microbiota‐derived metabolites, including short‐chain fatty acids, for their role in the gut‐brain axis. Finally, the effect of potential confounders, such as drug intake, may be explored by future research. The latter could take longitudinal or mechanistic approaches to determine the causative nature of the association between the gut microbiota and the brain in the present study.

Despite these limitations, this study boasts the strength of having predicted the value of a specific voxel by simultaneously considering the RA of all microbial families and controlling for other microbiota‐related variables.

To sum up, our study identified a brain structural biomarker corresponding to the gut bacterial ecosystem in a relatively large adult cohort. We devised a novel approach for analyzing multimodal brain MRI and gut microbiota data, including multiple tests due to the vast number of brain voxels. This innovative analytical strategy may help establish microbiome‐linked brain biomarkers in certain health disorders. Such biomarkers are crucial for studying the effects of long‐term dysbiosis on the brain and its associated factors. Researchers can gain valuable insights into the impact of altered gut microbiota on the brain and its related outcomes. This knowledge paves the way for future investigations into the gut microbiome's intricate relationship with brain health.

## Disclosure statement

V.P. has received payment honoraria for a master lecture from the EVES‐Conselleria de Sanitat Universal I Salut Pública, and support for attending to the XXIX Congress SEM from the University of Valencia. C.C. has received support for meeting attendance from Merch, BMS, Sanofi, and Novartis.

## Author contributions

Conception and design of the study: O.C., J.P., J.G., L.R., J.R., and J.M.F.R. Acquisition, analysis, and interpretation of data: G.B., C.B., M.A., C.C., A.M., V.P., and A.M. Drafting the manuscript or figures: O.C., J.M.F.R. and J.R. All authors discussed the results and contributed to review the final article.

## Supporting information


**Figure S1.** Relationship between the relative abundance of microbiota families and T2 values in the regions reported in Table 2.
**Figure S2.** Relationship between the relative abundance of microbiota families and axial diffusivity (AD), mean diffusivity (MD), and radial diffusivity (RD) diffusion tensor imaging (DTI)–related values in the regions reported in Table 2.
