# Supplementary material for: Unraveling the gut‐brain connection: The association of microbiota‐linked structural brain biomarkers with behavior and mental health
Source: Psychiatry Clin Neurosci. 2024 Feb 29;78(6):339–46. doi: 10.1111/pcn.13655 (PMC11488601; doi:10.1111/pcn.13655)
Supplement: Supplementary file 1 — Figure S1. Relationship between the relative abundance of microbiota families and T2 values in the regions reported in Table 2. Figure S2. Relationship between the relative abundance of microbiota families and axial diffusivity (AD), mean diffusivity (MD), and radial diffusivity (RD) diffusion tensor imaging (DTI)–related values in the regions reported in Table 2. [file PCN-78-339-s001.docx]

Supplementary Material

**Multimodal structural brain biomarkers of the gut microbiome ecosystems**

O Contreras-Rodriguez, M Arnoriaga-Rodríguez, G Blasco, C Biarnés, J Puig, C Coll-Martinez, Gich J, V Pérez-Brocal, A Moya, J Radua*, JM Fernandez-Real^*^


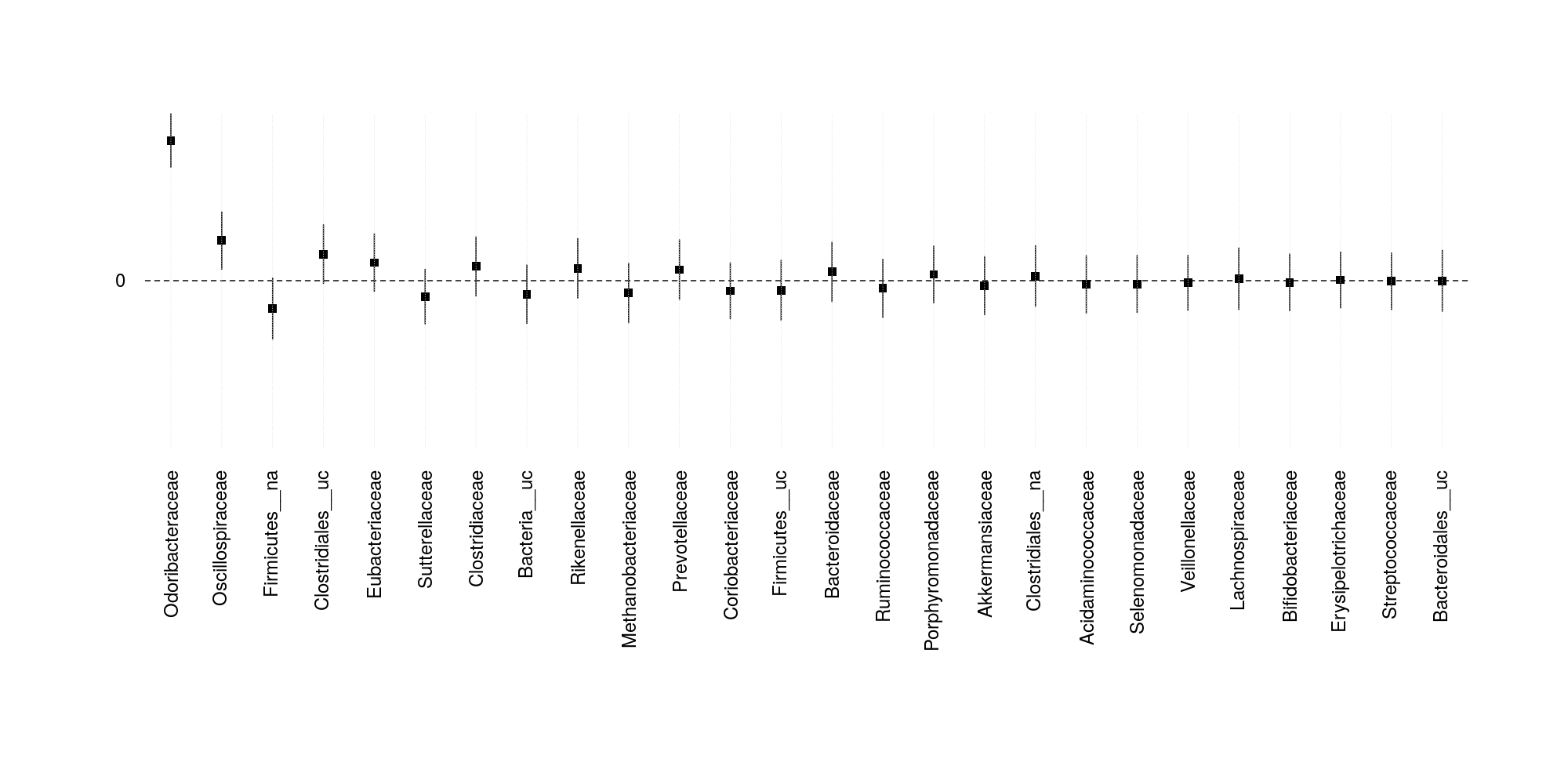

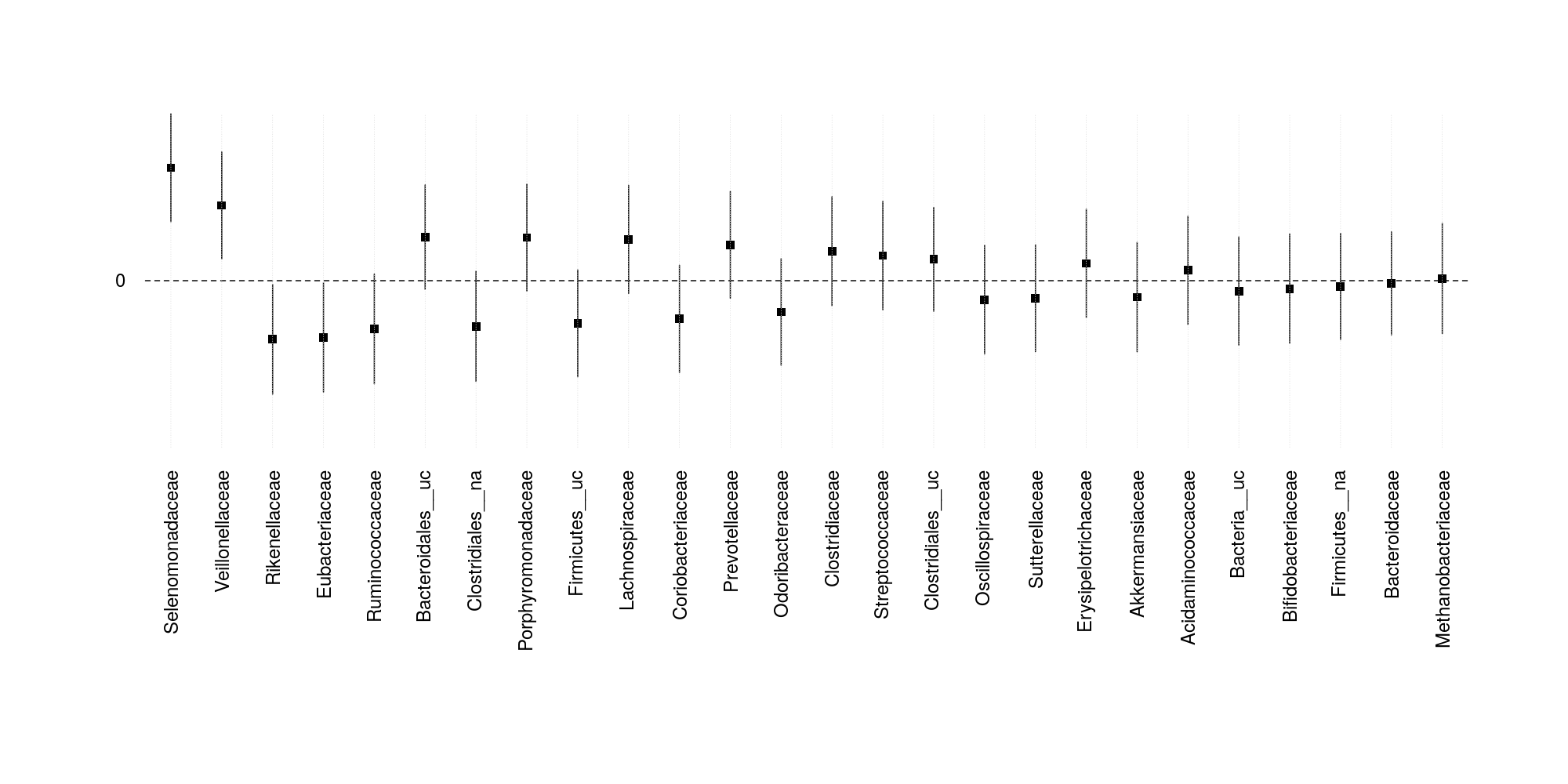

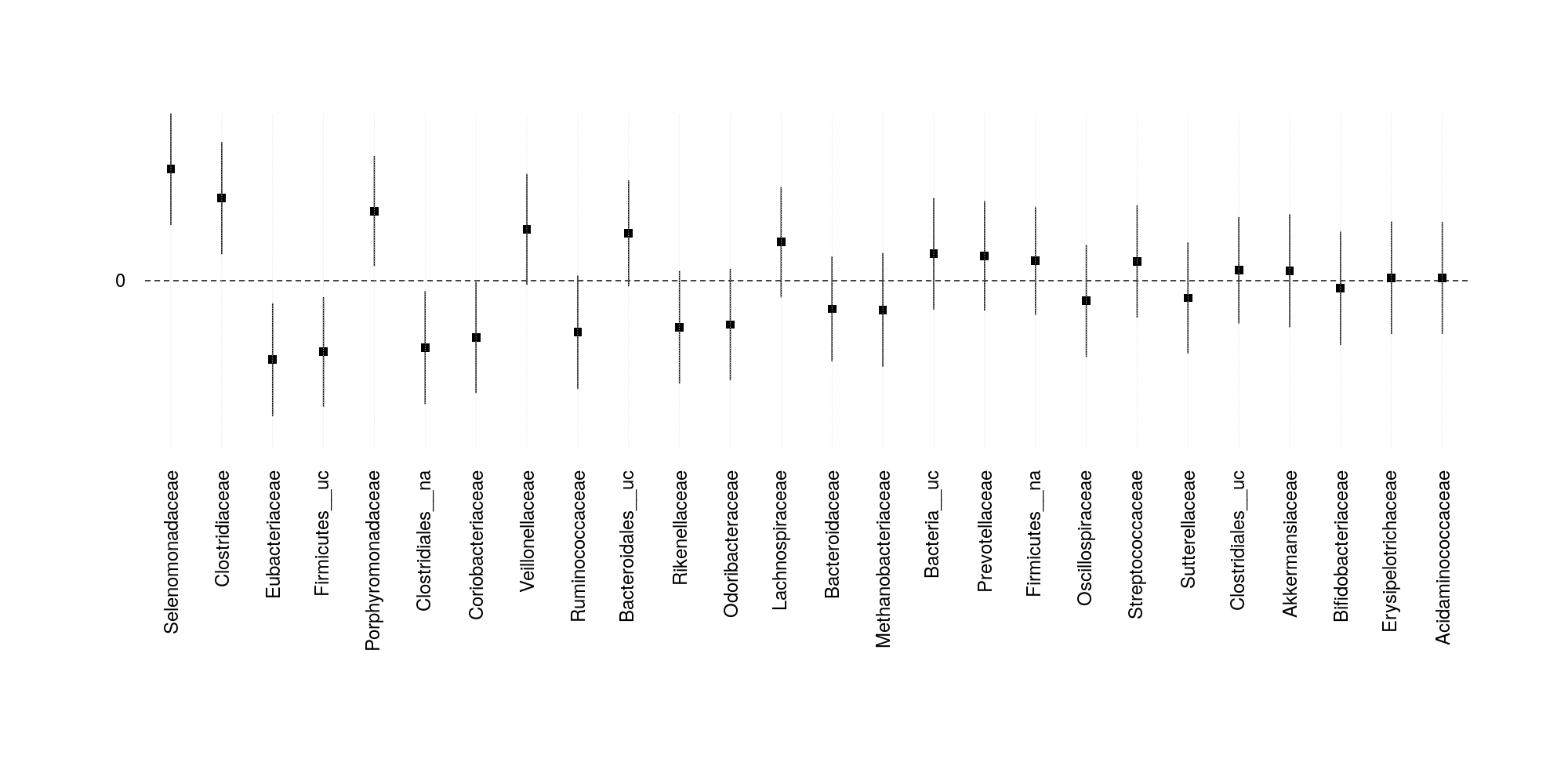
**Figure S1.** Relationship between the relative abundance of microbiota families and T2 values in the regions reported in Table 2.

Inferior occipital, x= -32, y= -82, z= -12

Cerebellum (Cr I-II), x= -28, y= -46, z= -38

Cerebellum (Cr I-II), x= 34, y= -48, z= -38


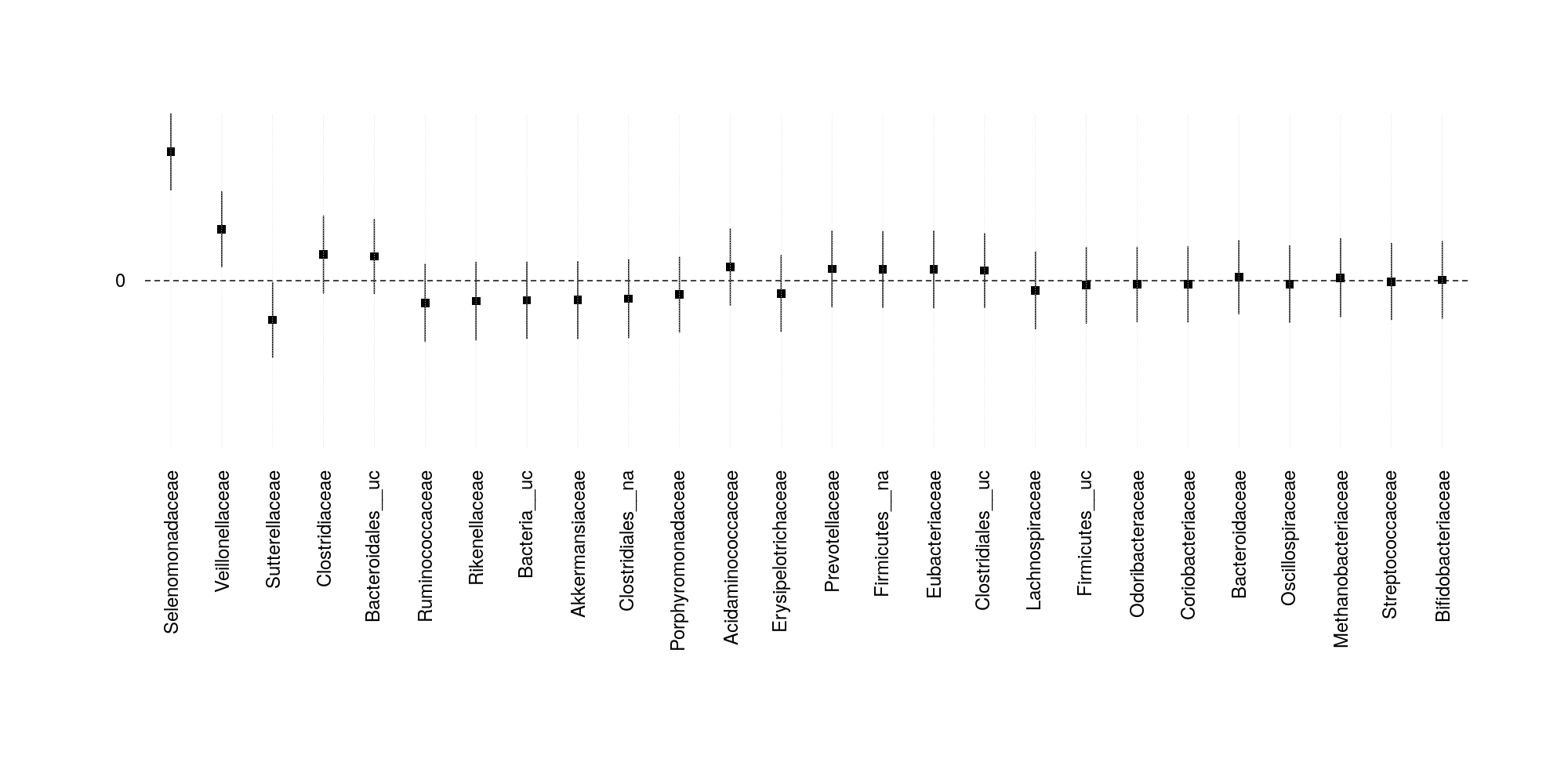

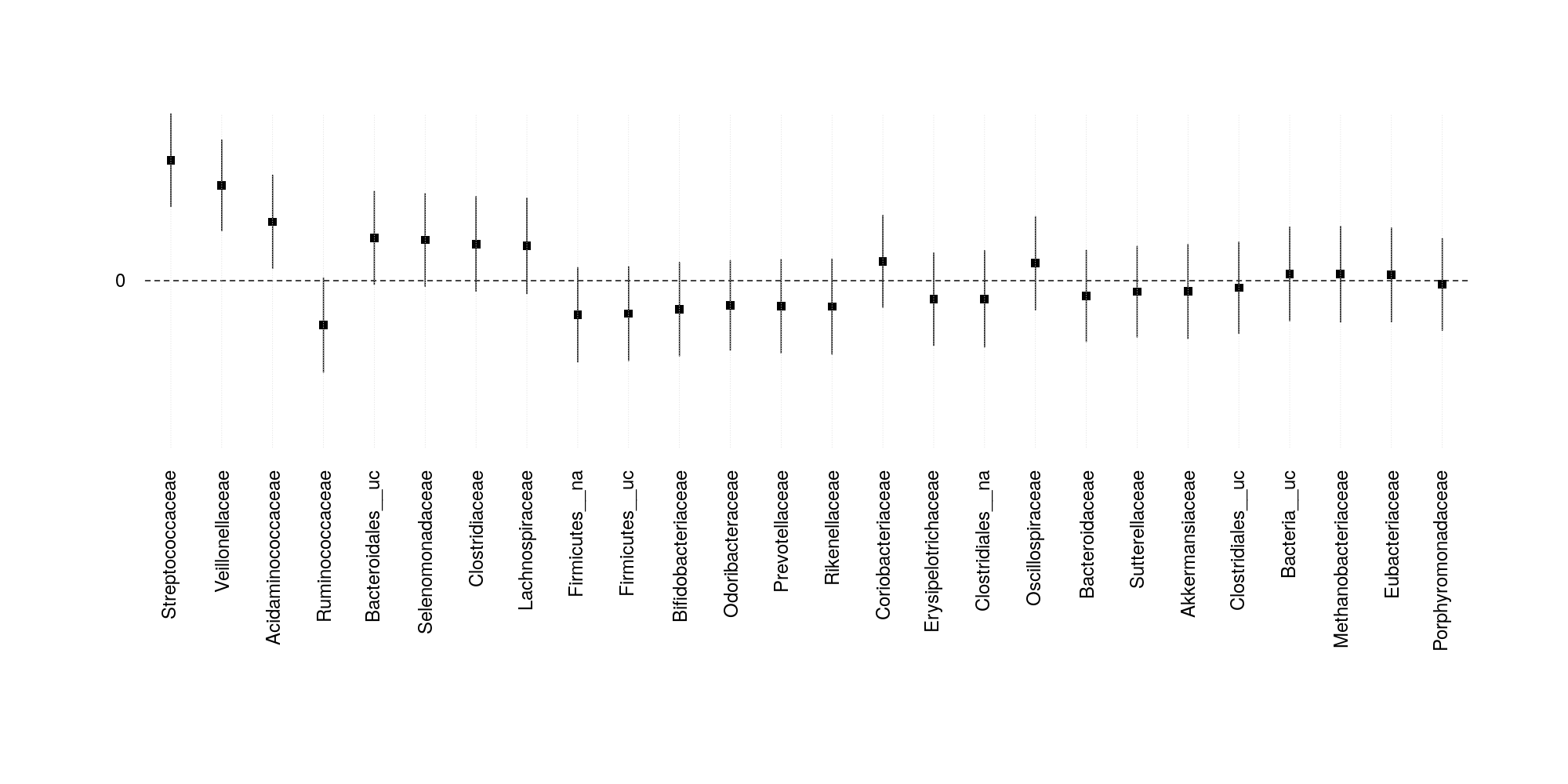

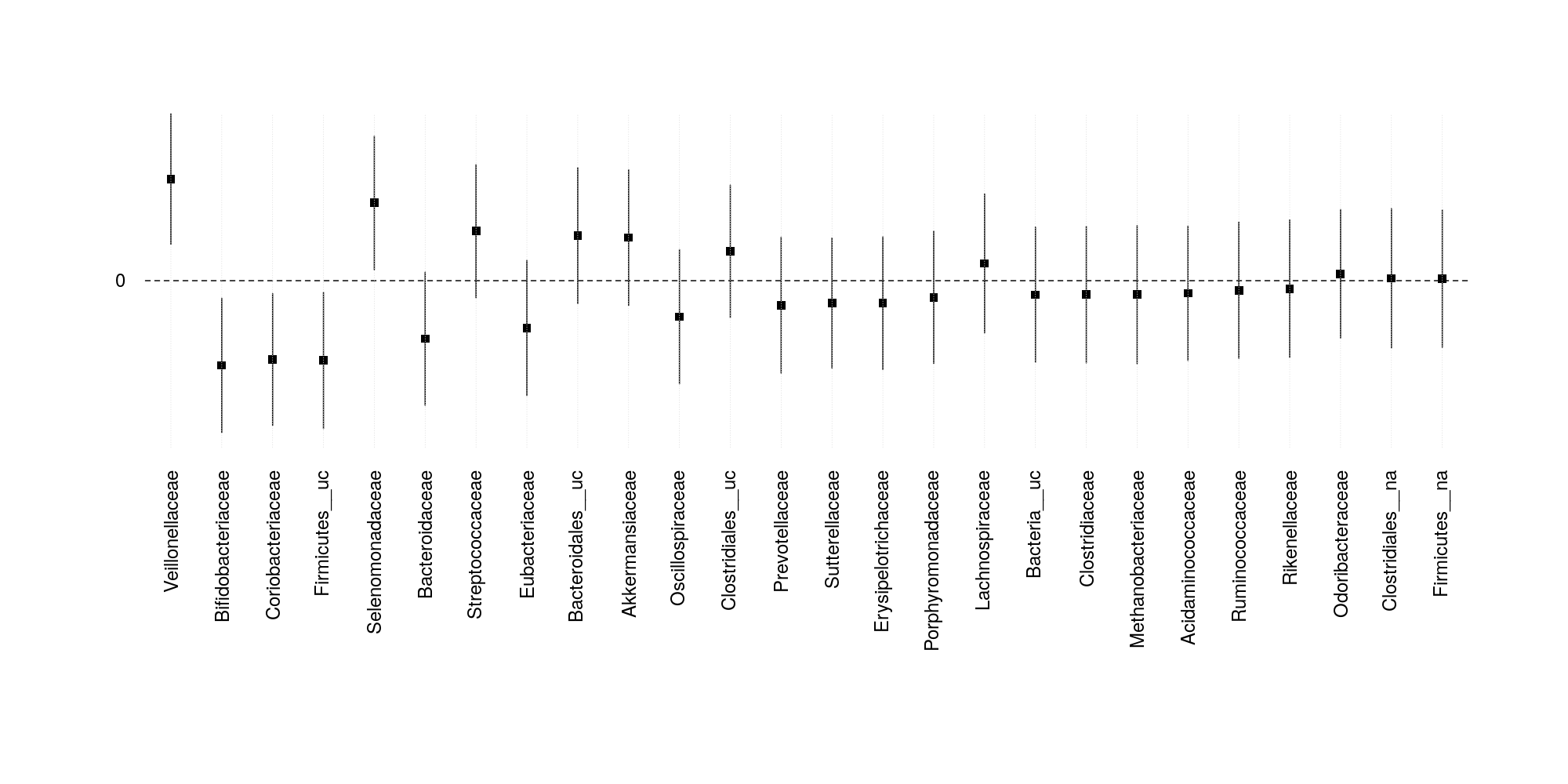
**Figure S1 (continues).** Relationship between the relative abundance of microbiota families and T2 values in the regions reported in Table 2.

Cerebellum (Cr I-II), x= -46, y= -56, z= -36

Medial gyrus rectus, x= 0, y= 44, z= -24

Fusiform gyrus, x= 36, y= -34, z= -30


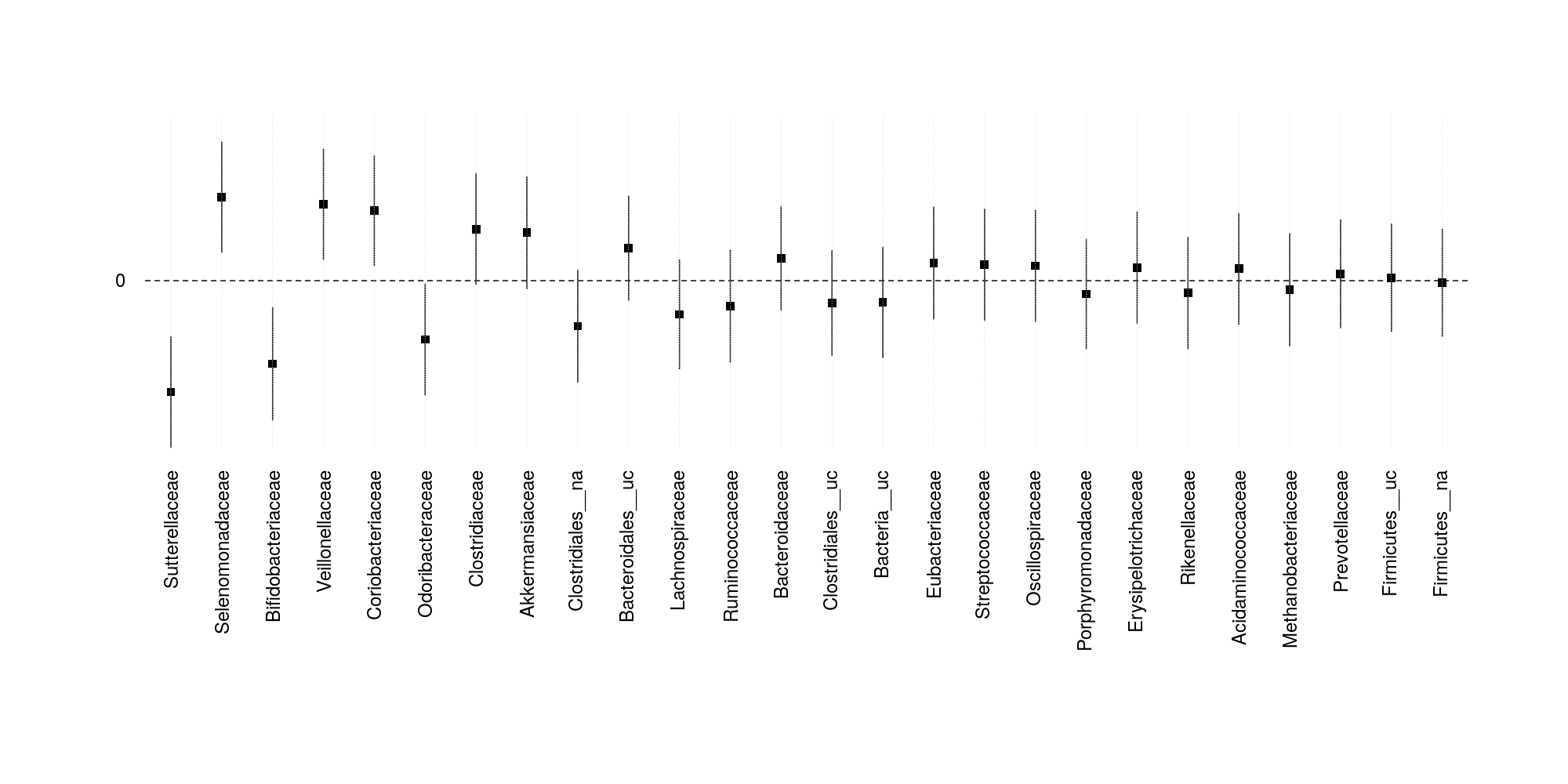

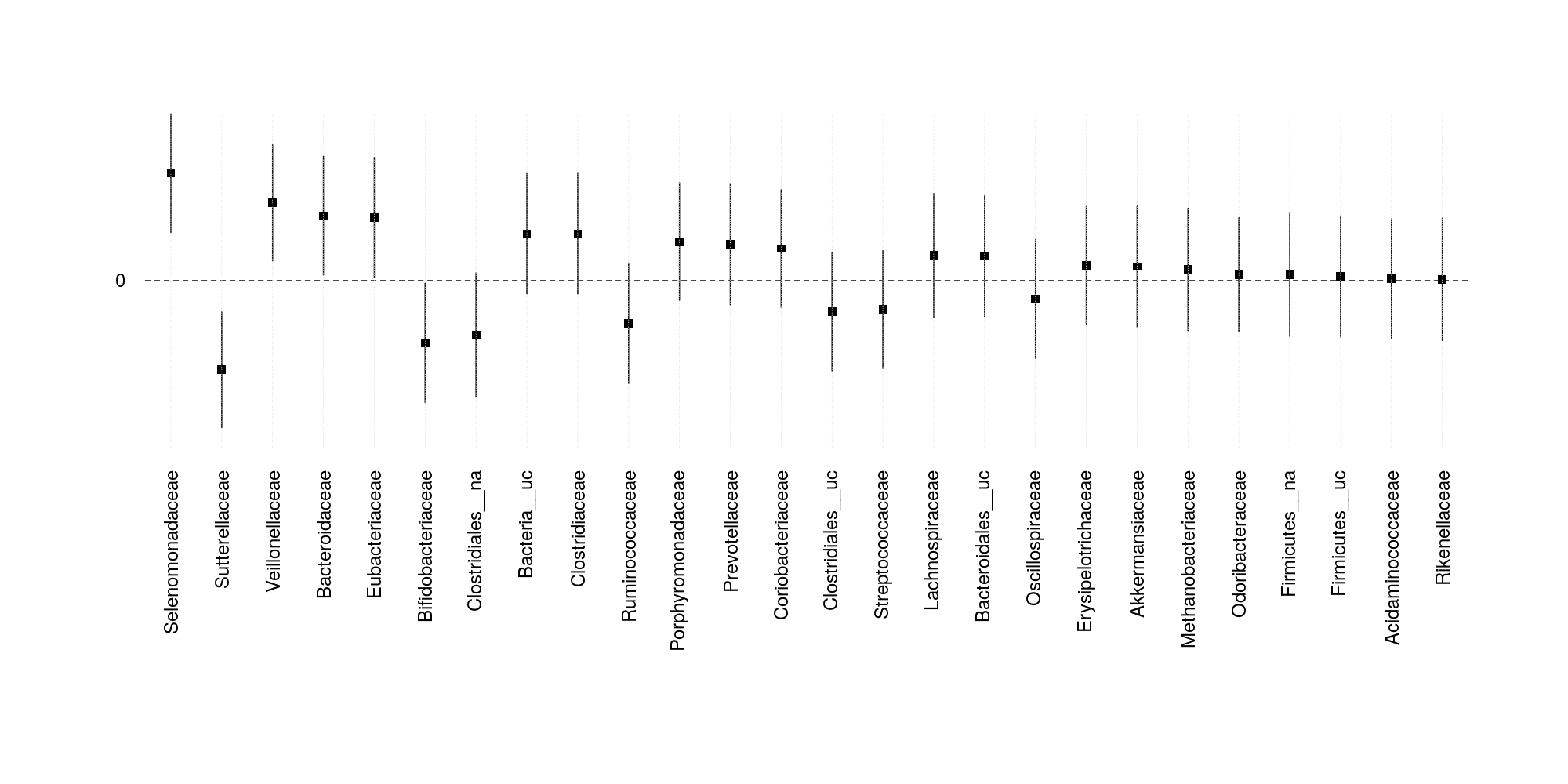
**Figure S2**. Relationship between the relative abundance of microbiota families and axial (AD), mean (MD) and radial (RD) DTI-related values in the regions reported in Table 2.

AD, Inferior frontal gyrus/insula, x= -48, y= 14, z= 6

AD, Middle Frontal gyrus, x= 56, y= 22, z= 40

AD, Dorsomedial frontal cortex, x= -4, y= 26, z= 50


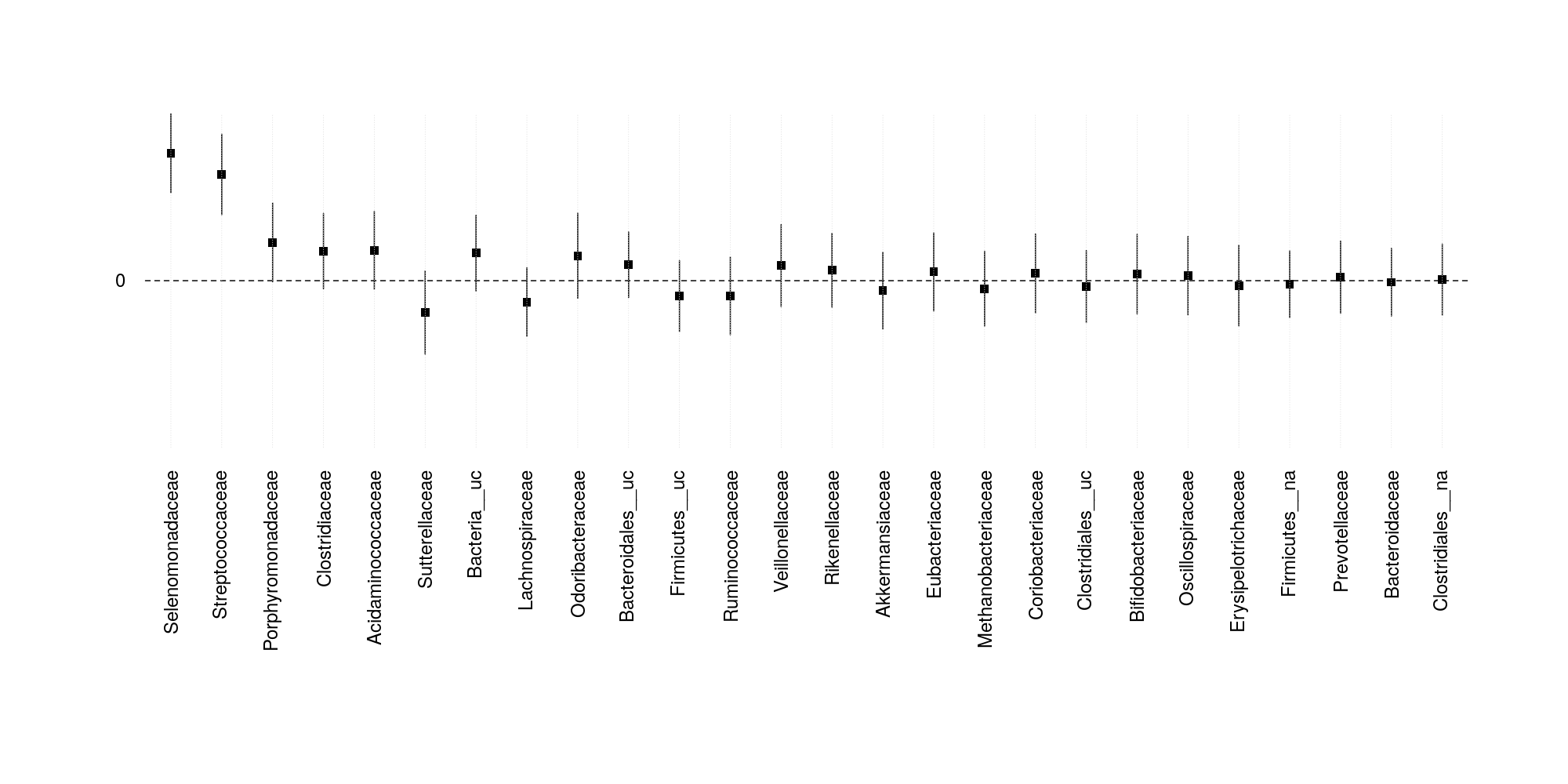


**Figure S2 (continues)**. Relationship between the relative abundance of microbiota families and axial (AD), mean (MD) and radial (RD) DTI-related values in the regions reported in Table 2.


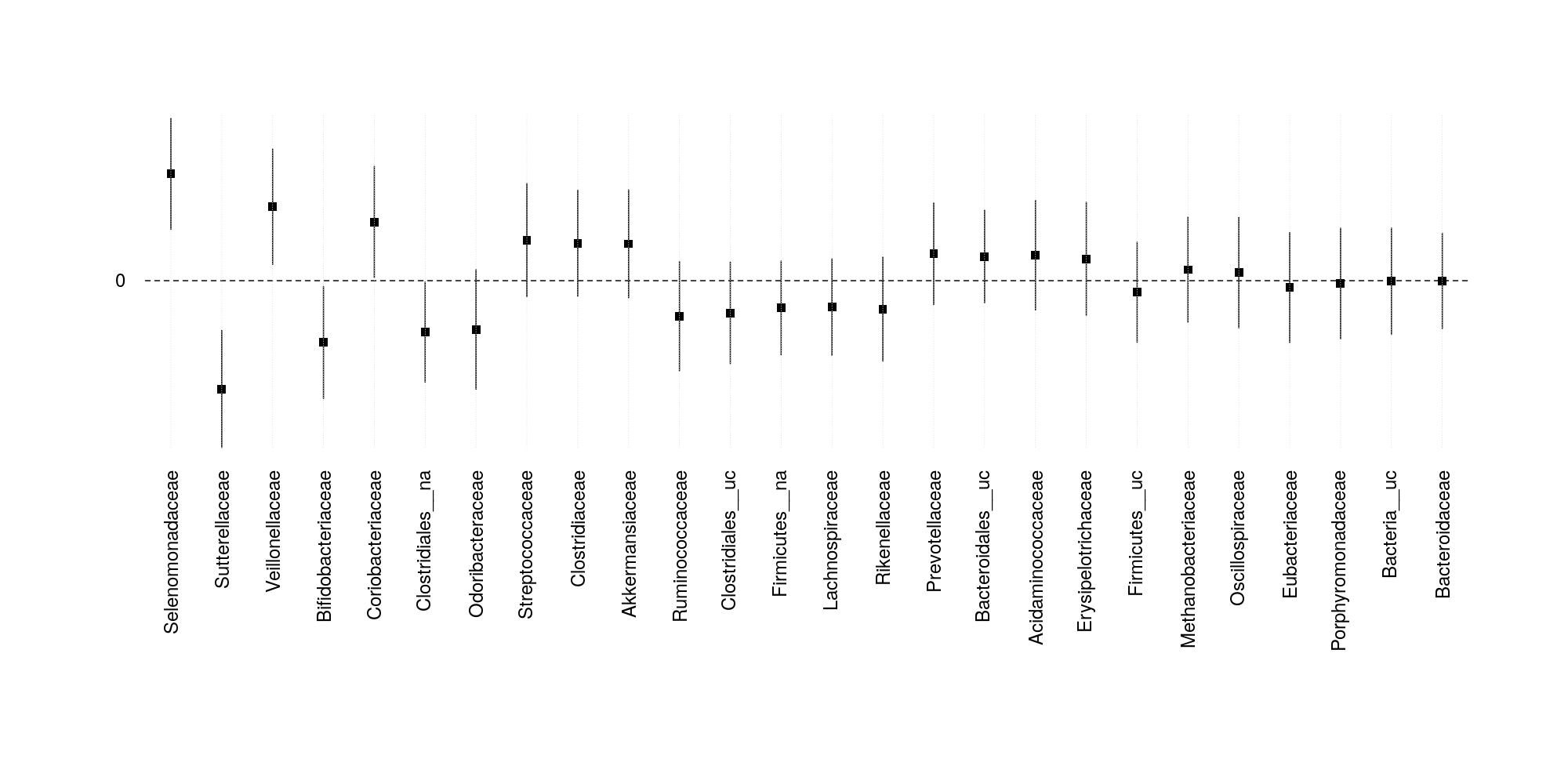

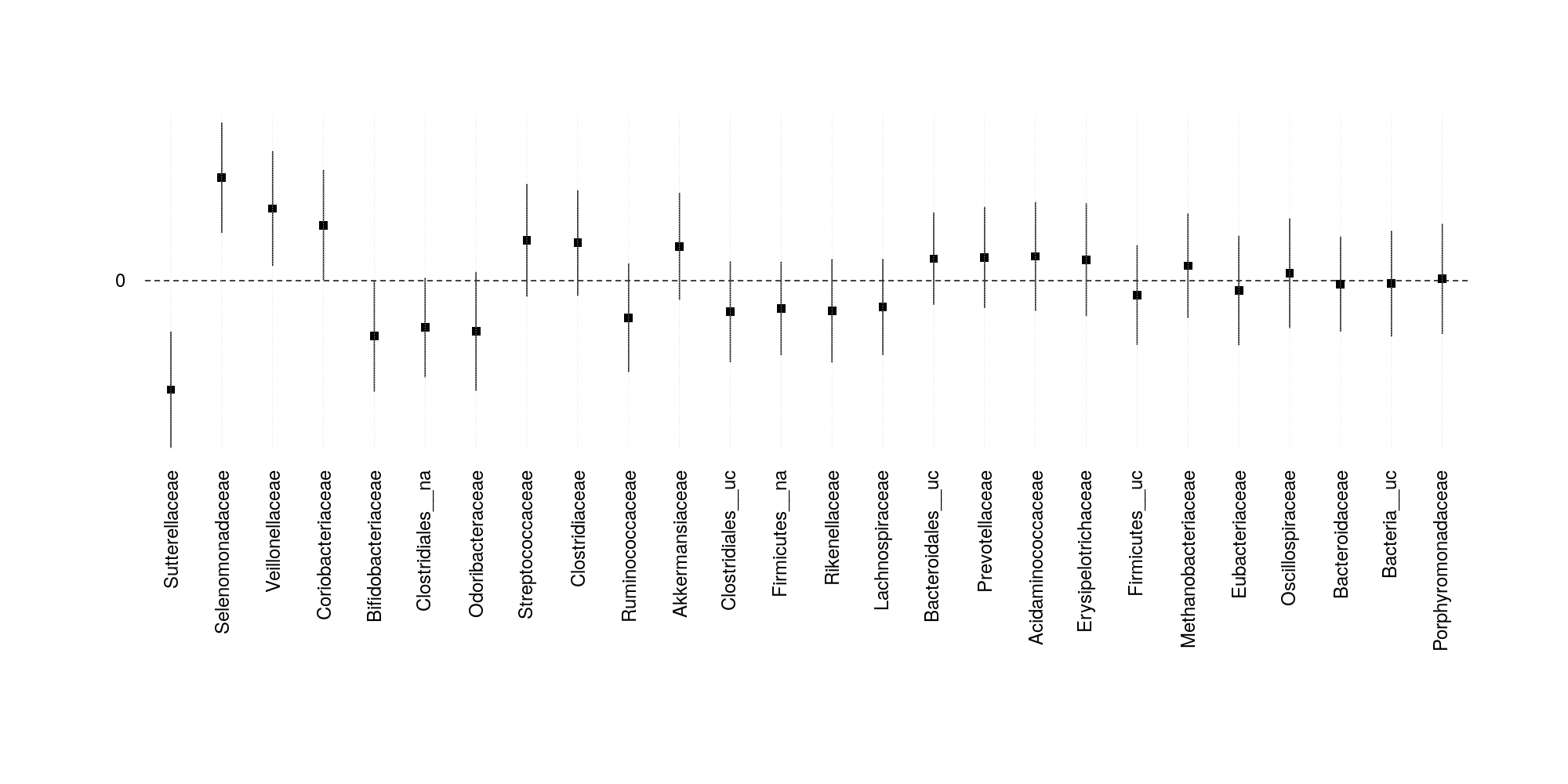

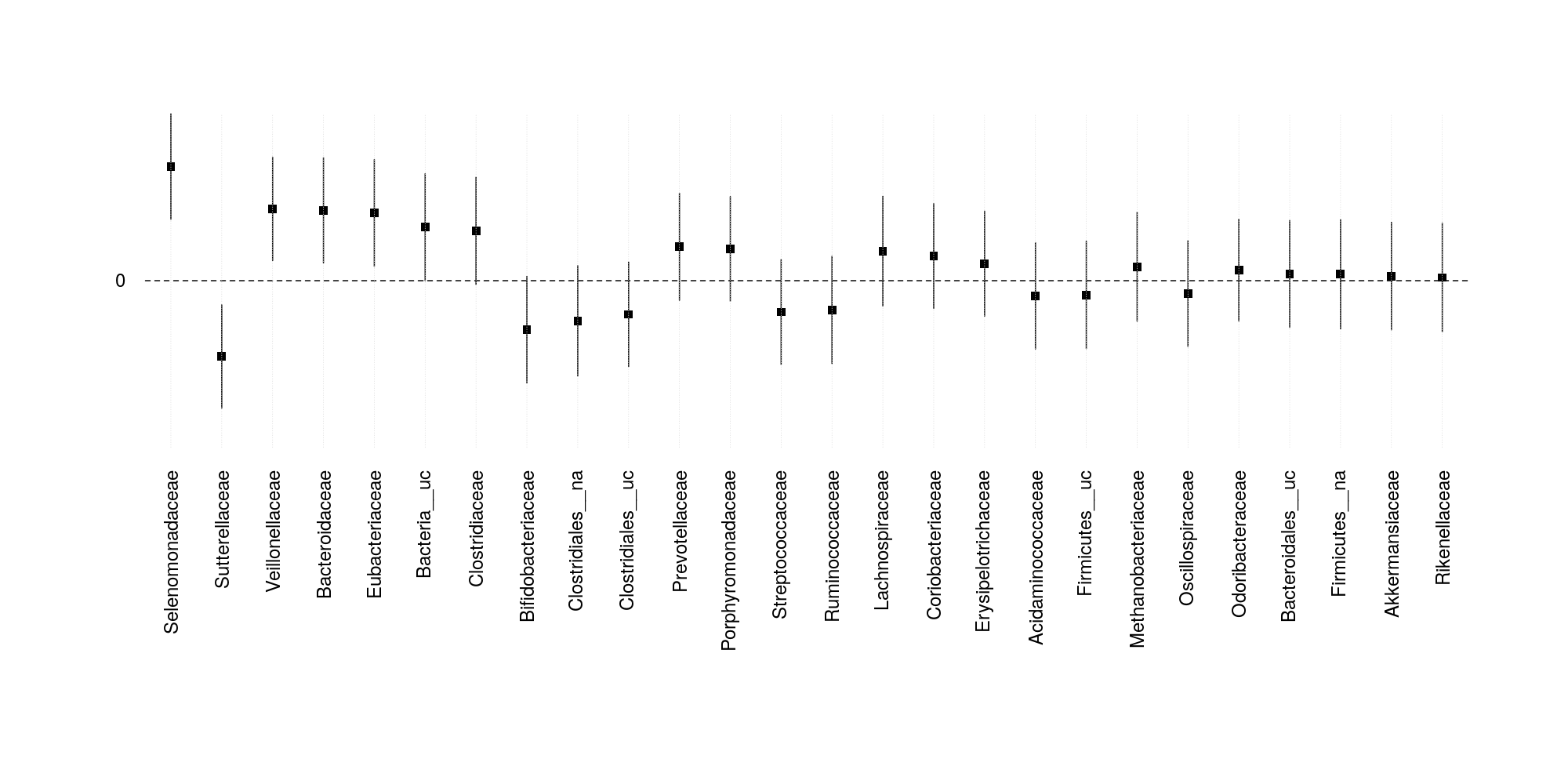


RD, Middle frontal gyrus, x= 56, y= 22, z= 40

MD, Middle frontal gyrus, x= 56, y= 22, z= 40

MD, Dorsomedial frontal cortex, x= -4, y= 28, z= 52


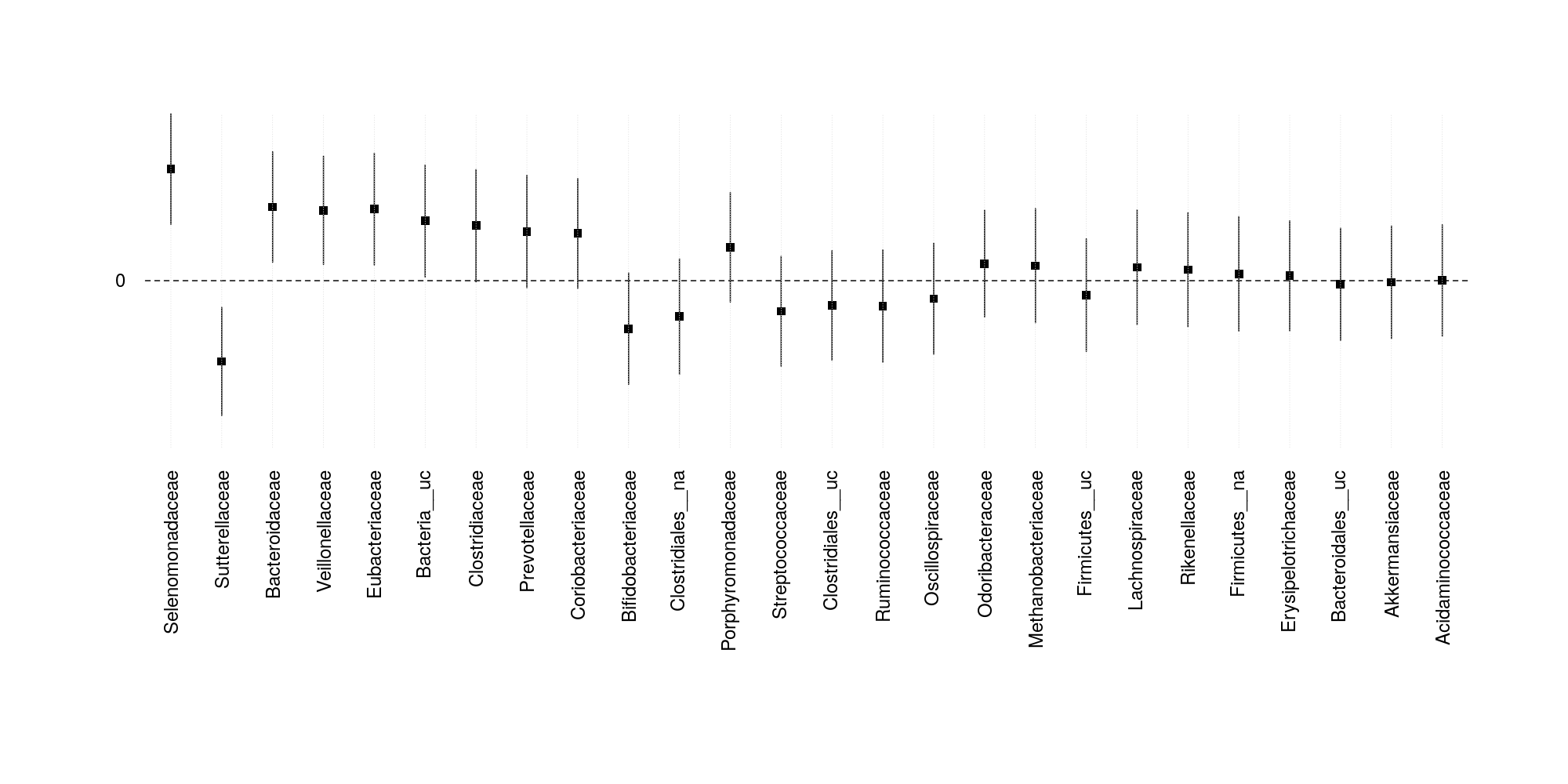
**Figure S2 (continues)**. Relationship the between abundance concentrations of microbiota families and axial (AD), mean (MD) and radial (RD) DTI-related values in the regions reported in Table 2.

RD, Dorsomedial frontal cortex, x= -4, y= 28, z= 50
